# Chromosomal mapping of 5S rDNA in two species of the genus *Acanthocephalus* (Echinorhynchida)

**DOI:** 10.1007/s00436-024-08381-0

**Published:** 2024-10-29

**Authors:** Martina Orosová, Anna Marková

**Affiliations:** 1 Institute of Parasitology, Slovak Academy of Sciences, Hlinkova 3, 040 01 Košice, Slovakia; 2 Department of Zoology, Faculty of Natural Sciences, Comenius University, Ilkovičova 6, 842 15 Bratislava, Slovakia

**Keywords:** Ribosomal genes, Fluorescence in situ hybridization, Multigene family, Acanthocephala

## Abstract

Chromosomal mapping of 5S rDNA in two Acanthocephala species was performed for the first time using fluorescence in situ hybridization (FISH) with a 5S rDNA probe. The 5S rDNA PCR products from the genomes of both species were sequenced and aligned and an identical 141 bp long coding region was determined. The same patterns of 5S rDNA gene cluster distribution were observed, with FISH signal restricted to a single autosomal chromosome pair. A preference for distal positioning on the chromosomes (subtelomeric position) was observed in both species. In addition, two-color FISH was performed to examine the mutual positions of 5S and 18S rDNA on the chromosomes. Our knowledge of the organization of the Acanthocephala genome is extremely limited and its chromosomes are poorly studied. Any new information about the location of chromosomal markers as important features of the respective karyotype may be useful in solving evolutionary questions.

## Introduction

Among the various multigene families in eukaryotic genomes, the highly conserved ribosomal DNA repeats (major 45S and minor 5S rDNA) are the most studied chromosomal markers since the introduction of fluorescence in situ hybridization (FISH) in animal and plant cytogenetics. These units are relatively stable and have proven to be useful molecular cytogenetic markers for the identification of chromosomes, for studies of genetic diversity within species and for understanding genome evolution. Both the 45S rDNA (18S, 5.8S and 28S RNA genes) and the 5S rDNA are characterized by concerted evolution (Nei and Rooney 2005), in which all members of the gene family are assumed to evolve in a concerted manner. The 5S rDNA cluster usually consists of several tandemly arranged arrays formed by approximately 120 nucleotides long coding sequences and variable non-transcribed spacer (NTS) sequences (Ciganda and Williams 2011) and can occur at any position on the chromosomes. Chromosome mapping of 45S and 5S rDNA by FISH in different animal species showed either separate localization of these families on different chromosomes (Salvadori et al. 2023), or co-localization on the same chromosomes (Tigano et al. 2004), as well as co-localization of 45S and 5S rDNA in single-repeat units (García-Souto and Pasantes 2015). In addition to the most frequent occurrence of 5S rDNA on autosomes, there are also species in which 5S rDNA occurs on sex chromosomes and B chromosomes (http://www.animalrdnadatabase.com).

Although genetic research on Acanthocephala has accelerated in the last decade, comparative cytogenetics is still underdeveloped. Recent cytogenetic studies using FISH with repetitive DNA probes (rDNA and histone genes) showed that the 18S rDNA sequence is present on one chromosome pair (chromosome X) in *A. lucii* and on two autosomal pairs in *A. anguillae*. At the same time, a clustered arrangement of histone H3 genes was found on one autosomal pair in *A. lucii*, while these sequences are scattered on all chromosomes in *A. anguillae* (Orosová et al. 2023; Marková et al. 2024). In two morphologically very similar species, *Pomphorhynchus laevis* and *P. tereticollis*, different chromosomal positions of the rRNA genes were found (Bombarová et al. 2007). Nothing is known about chromosomal markers in other species of the order Echinorhynchida, not even within the entire Acanthocephala. The aim of the present study was to perform a physical mapping of 5S rDNA in two acanthocephalan species in order to develop a new cytogenetic marker that will contribute to the understanding of chromosome evolution in this little-studied parasite group. In addition, we isolated and characterized the nucleotide sequences of 5S rDNA for the first time in the phylum Acanthocephala.

## Material and methods

### Biological material

Specimens of both species were collected from fish from the Zemplínska Šírava reservoir (48°47′09.0″N 21°57′20.5″E), eastern Slovakia (detailed information on the collected fish and parasite can be found in Orosová et al. 2023; Marková et al. 2024). Spread chromosome preparations were obtained following the "hot plate" technique described in Orosová and Špakulová (2018).

### Probe synthesis and labeling

Genomic DNA was extracted using the QIAamp® DNA mini Kit (QIAGEN, Hilden, Germany) according to the manufacturer’s instruction. Based on the Illumina paired-end sequencing data of *A. lucii* on the HiSeq 4000 platform at Novogene (HK) Co, Ltd (Hong Kong, China) (authors' unpublished raw dataset), new primers were designed for the amplification of 5S rDNA: Acanth5SF (GTGATCGAACGAGAACCGGT) and Acanth5SR (TCACAAACTTTCGCGCGTTA). Labeled 5S rDNA probe was obtained by standard PCR (35 cycles: initial denaturation step at 95 °C for 3 min; 35 cycles of 94 °C for 30 s, 59 °C for 30 s and 72 °C for 90 s; final extension at 72 °C for 3 min) with dNTP mix containing 0.35 mM biotin-16-dUTP (Roche Diagnostics). FISH was performed following the protocol described in Orosová et al. (2023). The hybridization signals of the biotin-labeled probes were detected with Cy3-conjugated streptavidin (Jackson ImmunoRes. Labs. Inc., West Grove, PA, USA) and amplified with biotinylated anti-streptavidin (Vector Labs. Inc., Burlingame, CA, USA), which in turn was detected with Cy3-conjugated streptavidin. Dual color FISH was performed to determine the mutual position of the 18S and 5S rDNA clusters on chromosomes. The 18S rDNA probes for both analysed species were labeled with biotin-16-dUTP by nick translation procedure and the 5S rDNA probes with digoxigenin-11-dUTP (Roche Diagnostics, Mannheim, Germany) by PCR. The DIG-labelled probe was detected using anti-digoxigenin-FITC (Sigma-Aldrich). Slides were analyzed using a LEICA DM 4000 B combined light and fluorescence microscope equipped with a DFC 450 C digital camera. Images were captured separately for each fluorescent dye, then pseudocolored and merged using Adobe Photoshop, version 7.0. In addition, the correctness of the primers developed in our work was tested by PCR with the DNA of various Acanthocephala species (*Bolbosoma vasculosum*, *Pallisentis rexus*, *Rhadinorhynchus laterospinosus*) deposited in our laboratory (provided by Daniel Barčák).

### Sequencing

Amplified products of 5S rDNA were run out on a 1.5% agarose gel, bands/fragments (approximately 300 bp) were dissected from gel, purified using the Wizard SV Gel and PCR Clean-Up System (Promega) according to manufacturer’s instruction and sequenced (SEQme, Dobříš, Czech Republic).

## Results and discussion

**Fig. 1:**
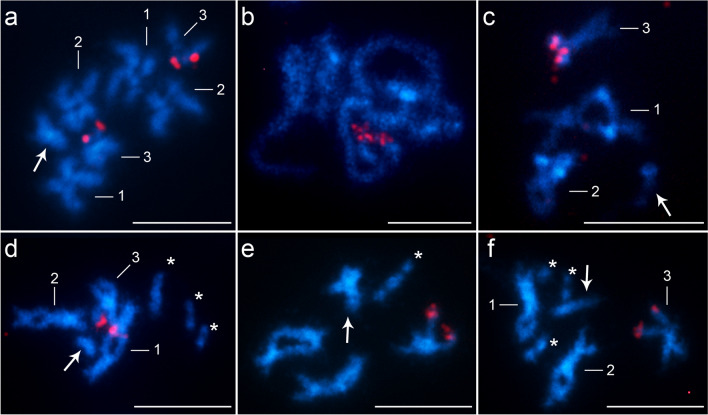
FISH with the 5S rDNA probe (red) on chromosomes of *Acanthocephalus anguillae* (**a**-**c**) and *A. lucii* (**d**-**f**). **a** Mitotic metaphase. **b** Pachytene nucleus. **c** Diplotene nucleus. **d**-**f** Male diplotene with B chromosomes. Arrows indicate X chromosomes and asterisks indicate B chromosomes. Chromosomes were counterstained with DAPI. Bar = 10 µm

**Fig. 2:**
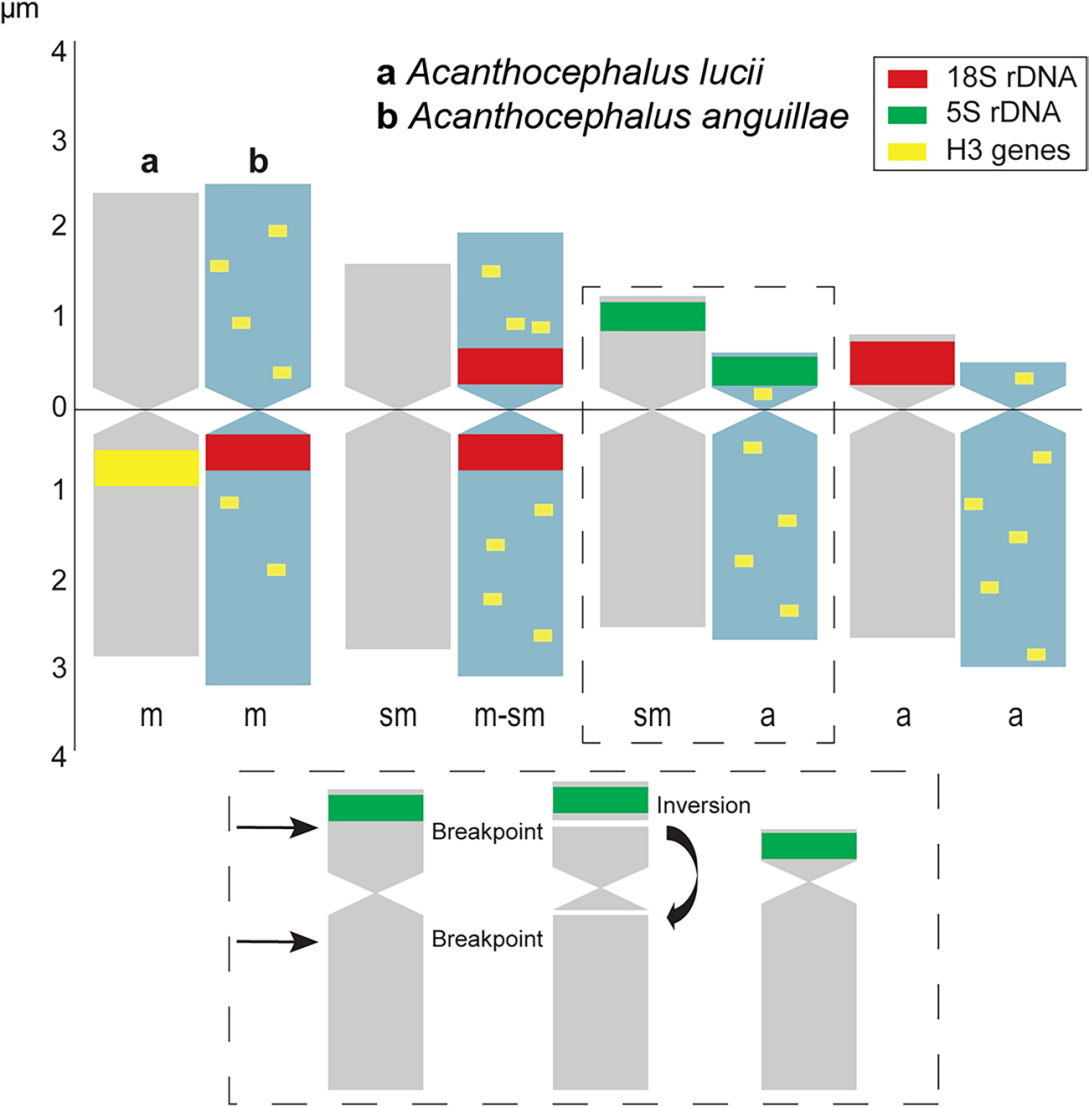
Comparison of idiograms of chromosomes of (**a**) *Acanthocephalus lucii* and (**b**) *A. anguillae* constructed from data on absolute length (µm) with a schematic interpretation of hypothetical pericentric inversion of submetacentric chromosome No. 3 in *A. lucii* to produce an acrocentric chromosome No. 3 found in *A. anguillae*. a – acrocentric; m – metacentric, sm – submetacentric chromosome pair

The results of our present work are part of a larger project in which we are devoting ourselves to a detailed karyological analysis of representatives of the parasite group Acanthocephala. The karyotypes of the studied species *A. lucii* and *A. anguillae* were determined in our recently published work (Orosová et al. 2023; Marková et al. 2024); 2n = 7/8 (male/female); n = 1 m + 1 m-sm + 1a + 1a (X) in the former and 2n = 7/8 n = 1 m + 2sm + 1a (X) in the latter. The present work provides for the first time the nucleotide sequence and chromosomal location of the 5S rDNA loci in two species of the genus *Acanthocephalus*. In the present study, one cluster of minor rDNA was detected at a single locus in both taxa (Fig. 1), located at a distal (subtelomeric) site on chromosome No. 3. In *A. anguillae*, the 5S rDNA site was located on the short arm of the acrocentric chromosome pair No. 3 (3pter) (Fig. 1a-c) and in *A. lucii* this site was located on the short arm of the submetacentric chromosome pair No. 3 (3pter) (Fig. 1d-f). The karyotypes of these two studied species are morphologically very similar and the basic characteristics (chromosome and relative length) of the corresponding chromosome pairs differ only slightly (Fig. 2). The main and only noticeable difference is the morphology of the third pair of chromosomes, which is submetacentric in the karyotype of *A. lucii*, but acrocentric in *A. anguillae* (Orosová et al. 2023; Marková et al. 2024). This pair No. 3 carries the 5S rDNA genes in subtelomeric position, which is considered as highly variable domain at the end of a chromosomal arm. These karyological data lead to the conclusion that chromosome No. 3 was involved in the changes in the course of evolution. Even on the basis of classical chromosome staining, it is evident that the genomes of *A. lucii* and *A. anguillae* diverged through intrachromosomal rearrangements that change the morphology but not the number of chromosomes. The last phylogenetic tree of these species, obtained from 18S rDNA sequences (Lisitsyna et al. 2023), showed that *A. lucii* is evolutionarily older than *A. anguillae*. All these data thus provided us with a conclusive “inversion” hypothesis that alters chromosomal morphology but not chromosome number, as shown schematically in Fig. 2. A pericentric inversion of submetacentric chromosome No. 3 would result in a shift of the centromere from the middle to a terminal position and leave the segment containing the rDNA cluster in the same location. The species are often differentiated by chromosomal rearrangements such as translocations and inversions (Huang and Riesenberg 2020). Previous studies, although very sporadic in this group of parasites (see summary table S1 in Orosová et al. 2023), showed a high karyotypic similarity in terms of number of chromosomes, but variability was observed in terms of morphology and distribution of 18S rDNA on chromosomes, suggesting that chromosomal rearrangements are quite common in the order Echinorhynchida. Chromosomal inversions, more often pericentric than paracentric, are widespread in the plant and animal kingdoms, as cytogenetic techniques have shown (García-Souto et al. 2017; Hooper and Price 2017), and play a significant role in speciation processes. Their important role in speciation has also been suggested in two Acanthocephala species of the genus *Pomphorhynchus* with respect to 18S rDNA position variation (Bombarová et al. 2007).

**Fig. 3:**
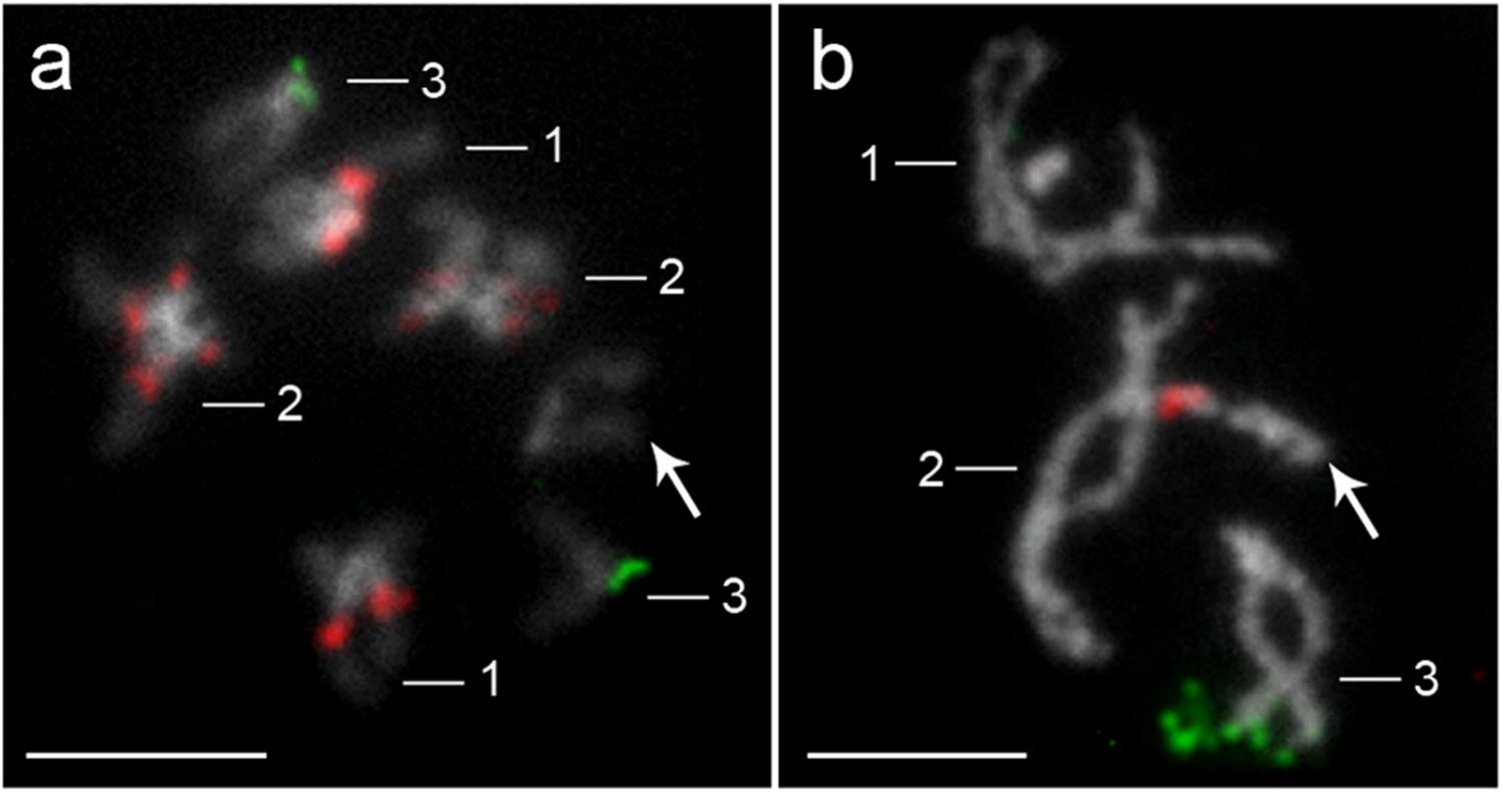
Two-color FISH with 18S rDNA (red) and 5S rDNA (green) probes on chromosomes of *Acanthocephalus anguillae* (**a**) and *A. lucii* (**b**) males. Arrows indicate X chromosomes. Chromosomes were counterstained with DAPI. Bar = 10 µm

Two-color FISH precisely localized the mutual position of two rDNA families (18S and 5S rDNA) on chromosomes and showed their separate location, on different chromosomes (Fig. 3). Investigated species originated from highly polluted water reservoir, with B chromosomes detected in their karyotypes, but no 5S rDNA FISH positive signal was found on B chromosomes (Fig. 1).

**Fig. 4:**
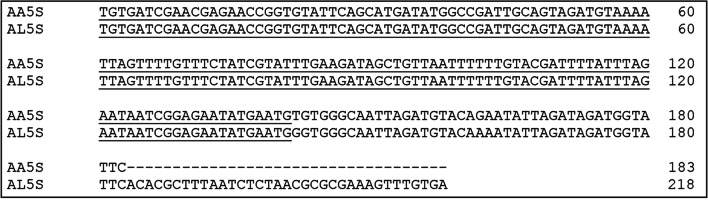
5S rDNA nucleotide sequences of *A. anguillae* (AA5S) and *A. lucii* (AL5S). The black line shows identical 141 bp long coding region

Based on the analysis of genome sequencing data of *A. lucii* (authors' personal data), the sequence of the repeat unit with a length of 784 bp was found to have after searches using BLAST an average degree of identity (94%) with the 5S rDNA sequence of *Schistocerca gregaria*. PCR amplification with primers newly designed for this sequence (Acanth5SF, Acanth5SR) resulted in a band around 300 bp for both analyzed species. After sequencing, the length of single repeat of 5S rDNA was found to be 218 bp long in *A. lucii* and 183 bp long in *A. anguillae* (Fig. 4). The obtained 5S rDNA nucleotide sequences of *A. lucii* and *A. anguillae* shared a highly conserved gene (100% identity) of 141 bp long coding sequence. The sequences were deposited in GenBank under accession numbers for *A. lucii* PP928417 and for *A. anguillae* PP931991.

The 5S rDNA nucleotide sequence results are preliminary and more species should be analyzed, but once these data are more robust, they may contribute to the systematics of the Acanthocephala group. The reliable identification of individual chromosomes in a species karyotype is the basis for successful comparative cytogenetic research. FISH signals on chromosomes are good markers for the correct identification of chromosomes and the pairing of homologs. By using 5S and 18S rDNA probes, it is now possible to consistently identify all autosomal chromosomes in the genome of *A. anguillae*, and the use of 5S rDNA as a chromosomal marker resolves the difficult pairing of homologous chromosomes #2 and #3, which are both submetacentric pairs in the karyotype of *A. lucii.* Recently, nucleotide/sequencing data of Acanthocephala have been increasing and are being incorporated into integrative taxonomic studies of Acanthocephala at the species/genus level (Perrot-Minnot et al. 2023). However, the inclusion of some cytogenetic data, such as information on chromosome rearrangements and their role in the speciation process, in these studies could also be very useful.

## Data Availability

No datasets were generated or analysed during the current study.

## References

[CR1] Bombarová M, Marec F, Nguyen P, Špakulová M (2007) Divergent location of ribosomal genes in chromosomes of fish thorny-headed worms, *Pomphorhynchus**laevis* and *Pomphorhynchus**tereticollis* (Acanthocephala). Genetica 131:141–149. 10.1007/s10709-006-9124-3 17143651 10.1007/s10709-006-9124-3

[CR2] Ciganda M, Williams N (2011) Eukaryotic 5S rRNA biogenesis. Wiley Interdiscip Rev RNA 2:523–33. 10.1002/wrna.74 21957041 10.1002/wrna.74 PMC3278907

[CR3] García-Souto D, Pasantes JJ (2015) Molecular cytogenetics in digenean parasites: linked and unlinked major and 5S rDNAs, B chromosomes and karyotype diversification. Cytogenet Genome Res 147(2–3):195–207. 10.1159/000442504 26680763 10.1159/000442504

[CR4] García-Souto D, Peréz-García C, Pasantes JJ (2017) Are pericentric inversions reorganizing wedge shell genomes? Genes 8(12):370. 10.3390/genes8120370 29215567 10.3390/genes8120370 PMC5748688

[CR5] Hooper DM, Price TD (2017) Chromosomal inversion differences correlate with range overlap in passerine birds. Nat Ecol Evol 1(10):1526–1534. 10.1038/s41559-017-0284-6 29185507 10.1038/s41559-017-0284-6

[CR6] Huang K, Riesenberg LH (2020) Frequency, origins, and evolutionary role of chromosomal inversions in plants. Front Plant Sci 18(11):296. 10.3389/fpls.2020.00296 10.3389/fpls.2020.00296 PMC7093584 32256515

[CR7] Lisitsyna O, Barčák D, Orosová M, Fan CF, Oros M (2023) Acanthocephalans of marine and freshwater fishes from Taiwan with description of a new species. Folia Parasit 70:021. 10.14411/fp.2023.021 10.14411/fp.2023.021 38167244

[CR8] Marková A, Orosová M, Marec F, Barčák D, Oros M (2024) Karyological study of *Acanthocephalus**lucii* (Echinorhynchida): the occurrence of B chromosomes in populations from PCB-polluted waters. Diversity 16:140. 10.3390/d16030140

[CR9] Nei M, Rooney AP (2005) Concerted and birth-and-death evolution of multigene families. Annu Rev Genet 39:121–152. 10.1146/annurev.genet.39.073003.112240 16285855 10.1146/annurev.genet.39.073003.112240 PMC1464479

[CR10] Orosová M, Špakulová M (2018) Tapeworm chromosomes: their value in systematics with instructions for cytogenetic study. Folia Parasit 65:001. 10.14411/fp.2018.001 10.14411/fp.2018.001 29528298

[CR11] Orosová M, Marková A, Zrzavá M, Marec F, Oros M (2023) Chromosome analysis and the occurrence of B chromosomes in fish parasite *Acanthocephalus**anguillae* (Palaeacanthocephala: Echinorhynchida). Parasite 30:44. 10.1051/parasite/2023045 37870409 10.1051/parasite/2023045 PMC10592040

[CR12] Perrot-Minnot MJ, Cozzarolo CS, Amin O et al (2023) Hooking the scientific community on thorny-headed worms: interesting and exciting facts, knowledge gaps and perspectives for research directions on Acanthocephala. Parasite 30:23. 10.1051/parasite/2023026 37350678 10.1051/parasite/2023026 PMC10288976

[CR13] Salvadori S, Deidda F, Carugati L, Melis R, Costa E, Sibiriu M, Coluccia E (2023) Chromosomal mapping of ribosomal clusters and telomeric sequences (TTAGG)_*n*_ in nine species of lobsters (Crustacea, Decapoda). Eur Zool J 90:443–453. 10.1080/24750263.2023.2217188

[CR14] Tigano C, Rocco L, Ferrito V, Costagliola D, Pappalardo AM, Stingo V (2004) Chromosomal mapping and molecular characterization of ribosomal RNA genes in *Lebias**fasciata* (Teleostei, Cyprinodontidae). Genetica 121:95–100. 10.1023/B:GENE.0000019931.89458.dc 15098742 10.1023/b:gene.0000019931.89458.dc

